# Navigating stem cell culture: insights, techniques, challenges, and prospects

**DOI:** 10.3389/fcell.2024.1435461

**Published:** 2024-11-11

**Authors:** Aleksandra Górska, Mateusz Trubalski, Bartosz Borowski, Adam Brachet, Sylwia Szymańczyk, Renata Markiewicz

**Affiliations:** ^1^ Department of Normal, Clinical and Imaging Anatomy, Medical University of Lublin, Lublin, Poland; ^2^ Students Scientific Association, Department of Normal, Clinical and Imaging Anatomy, Medical University of Lublin, Lublin, Poland; ^3^ Student Scientific Association, Department of Forensic Medicine, Medical University of Lublin, Lublin, Poland; ^4^ Department of Animal Physiology, Faculty of Veterinary Medicine, University of Life Sciences in Lublin, Lublin, Poland; ^5^ Occupational Therapy Laboratory, Chair of Nursing Development, Medical University of Lublin, Lublin, Poland

**Keywords:** stem, cells, culture, methods, pluripotency, perspectives

## Abstract

Stem cell research holds huge promise for regenerative medicine and disease modeling, making the understanding and optimization of stem cell culture a critical aspect of advancing these therapeutic applications. This comprehensive review provides an in-depth overview of stem cell culture, including general information, contemporary techniques, encountered problems, and future perspectives. The article begins by explaining the fundamental characteristics of various stem cell types, elucidating the importance of proper culture conditions in maintaining pluripotency or lineage commitment. A detailed exploration of established culture techniques sheds light on the evolving landscape of stem cell culture methodologies. Common challenges such as genetic stability, heterogeneity, and differentiation efficiency are thoroughly discussed, with insights into cutting-edge strategies and technologies aimed at addressing these hurdles. Moreover, the article delves into the impact of substrate materials, culture media components, and biophysical cues on stem cell behavior, emphasizing the intricate interplay between the microenvironment and cell fate decisions. As stem cell research advances, ethical considerations and regulatory frameworks become increasingly important, prompting a critical examination of these aspects in the context of culture practices. Lastly, the article explores emerging perspectives, including the integration of artificial intelligence and machine learning in optimizing culture conditions, and the potential applications of stem cell-derived products in personalized medicine. This comprehensive overview aims to serve as a valuable resource for researchers and clinicians, fostering a deeper understanding of stem cell culture and its key role in advancing regenerative medicine and biomedical research.

## Introduction

In the dynamic field of stem cell research, the evolution of culture methodologies plays a pivotal role in unlocking the vast therapeutic and scientific potential inherent in these remarkable cells. This article provides a comprehensive overview, synthesizing key findings from seminal studies to offer a nuanced exploration of general information, techniques, persistent challenges, and future perspectives in the domain of stem cell culture. The past decade has witnessed groundbreaking discoveries that have reshaped our understanding of stem cells (Chen et al., 2014). The work of Takahashi and Yamanaka (Takahashi and Yamanaka, 2013) introduced induced pluripotent stem cells (iPSCs), a transformative concept that facilitated the reprogramming of somatic cells into a pluripotent state, bypassing ethical concerns associated with embryonic stem cells (Aboul-Soud et al., 2021; Yamanaka, 2020; Karagiannis et al., 2019; Liu et al., 2020). Concurrently, the investigations furthered our understanding of human embryonic stem cells (hESCs), contributing to the establishment of robust culture protocols for these cells (Kobold et al., 2023). Advancements in stem cell culture techniques have been instrumental in harnessing the full potential of these cells for therapeutic applications. Research by Chen et al. (2019) emphasized the importance of three-dimensional (3D) culture systems, demonstrating their superiority in maintaining stem cell pluripotency and enhancing differentiation potential compared to traditional two-dimensional (2D) approaches (Ylostalo, 2020). A comparison of 2D and 3D cultures can be found in Table 1. Additionally, the recent article of Yu et al. (2023) showcased the integration of microfluidic platforms to precisely control the stem cell microenvironment, influencing cellular responses in unprecedented ways. However, the translation of stem cell research into clinical applications is not without its challenges (McKee and Chaudhry, 2017). There is the persistent issue of genetic instability in long-term cultures, necessitating a nuanced approach to mitigate risks associated with genomic alterations (Prieto Gonzalez, 2022). Furthermore, the intrinsic heterogeneity within stem cell populations is a major hurdle, demanding innovative strategies to enhance the homogeneity of cell populations for therapeutic efficacy (Mas-Bargues and Borrás, 2021; Wang et al., 2021a). Looking ahead, the article explores emerging perspectives and potential solutions to current challenges. Recent investigations showcase the integration of artificial intelligence (AI) and machine learning in optimizing stem cell culture conditions, promising data-driven insights for enhanced reproducibility and efficiency (Nosrati and Nosrati, 2023). Additionally, Li et al. (2018) discusses the transformative potential of stem cell-derived products in personalized medicine, providing a glimpse into a future where tailored therapeutic interventions are based on individual patient profiles (Li et al., 2018). This review aims to consolidate the wealth of knowledge offering a comprehensive and up-to-date exploration of stem cell culture to optimize methodologies, propelling the field towards its full potential in biomedical research.

**TABLE 1:** Comparison of 2D and 3D cultures (Raitanen et al., 2023; Souza et al., 2018; Ylostalo, 2020; Baharvand et al., 2006).

Criterion	2D cell culture system	3D cell culture system
Cell Structure	Monolayer of cells on a flat surface	Three-dimensional structures with cell aggregates
Culturing Environment	Flat surface (e.g., Petri dish)	Gel matrices, microspheres, or bioreactors
Cell Interactions	Limited, primarily surface interactions	Fully three-dimensional, more complex interactions
*In Vivo* Representation	Limited, less realistic	Better simulation of *in vitro* environment
Phenotypic Impact	May lead to altered cell properties	Better retention of *in vitro* cell properties
Metabolic Efficiency	Often less representative	Typically better, more akin to natural conditions
Applications	Rapid and cost-effective assays, basic research	Differentiation studies, therapies, tissue engineering
Technical Requirements	Lower, easier to use	Higher, requires more advanced technologies

## General information about stem cells

In the context of developmental ontogenesis, stem cells (SCs) can be broadly classified into two principal categories: embryonic stem cells (ESCs), isolated from the inner cell mass of blastocysts, and adult stem cells (ASCs), colloquially referred to as somatic stem cells (SSCs), distributed across various adult tissues (Slack, 2018; Gao et al., 2018). Embryonic stem cells (ESCs), characterized by their self-renewal capacity, embody pluripotency, originating from the inner cell mass (ICM) of developing blastocysts. Pluripotency signifies the singular cell’s capability to engender all cellular lineages throughout both embryonic and adult organismal development. Tissue-specific stem cells orchestrate the dynamic turnover of distinct tissue phenotypes in mammals and other metazoans (Young, 2011; Christopher and Baker, 2018). Their ubiquitous presence spans a diverse array of tissues, including but not limited to bone marrow (BMSCs), adipose tissue (ADSCs), dental pulp (DPSCs), blood (HSCs), amniotic fluid (AFSCs), umbilical cord (UCSCs), and a sundry of other tissues. Despite sharing a vernacular nomenclature, these classifications reveal myriad distinctions (Zeyu et al., 2023; Orticelli et al., 2021).

Segregated according to their differentiative potential, stem cells delineate into totipotent SCs, pluripotent SCs (PSCs), multipotent SCs, and unipotent SCs (Gao et al., 2018). Totipotent cells, represented by the zygote and early blastomeres, possess the exceptional ability to give rise to all cellular lineages, playing a central role in initiating organismal development (Maemura et al., 2021). Conversely, pluripotent cells, found in the inner cell mass of blastocysts, exhibit a broad capacity to generate all cell types, except those specific to the extraembryonic trophoblast lineage. This distinction underscores the critical roles these cell types play in the early stages of life, with totipotent cells guiding the foundational steps of complete organism formation, and pluripotent cells contributing to the differentiation of diverse cell types crucial for embryonic development (Cai et al., 2022; Malik and Wang, 2022; Sobhani et al., 2017). Multipotent stem cells manifest the ability to differentiate into all cell types from a single germ layer, while unipotent stem cells are defined by their competence to differentiate solely into one lineage (Kolios and Moodley, 2013). Oligopotent stem cells, distinguished by their unique capacity for self-renewal, demonstrate the remarkable ability to initiate and sustain two or more distinct lineages within a specific tissue. An exemplary illustration of oligopotent stem cells can be observed in hematopoietic stem cells, known for their versatility in differentiating into both myeloid and lymphoid lineages, thereby contributing to the diverse cellular composition of the blood (Cheng et al., 2020).

In the context of pulmonary biology, ongoing investigations propose a fascinating possibility: bronchoalveolar duct junction cells, identified as oligopotent, may serve as the originators of both bronchiolar epithelium and alveolar epithelium. This intriguing finding underscores the dynamic nature of oligopotent stem cells in orchestrating tissue-specific regeneration and underscores their role in maintaining the intricate cellular architecture of the respiratory system (Ilic and Polak, 2011).

The phenomenon of continuous regeneration serves as an intrinsic process orchestrated by stem cells, playing a pivotal role in maintaining homeostasis within multicellular organisms. Senescent, functionally specialized mature cells within adult organs are perpetually supplanted by newly generated cells (Lee and Hong, 2020). This cellular turnover process is notably heightened within the hematopoietic system, intestinal epithelium, or epidermis, in stark contrast to the more measured pace observed in skeletal muscles, liver, or cardiac tissues (Ratajczak et al., 2012). Tissues characterized by a high rate of cellular turnover, such as blood and gut epithelium, harbor populations of incessantly proliferating adult stem cells and progenitor cells, adept at generating differentiating progeny. Additionally, many tissues maintain populations of stem cells in a quiescent state, either to provide support for cycling progenitors or to serve as a reserve for potential tissue injuries (de Morree and Rando, 2023). A pivotal prerequisite for a stem cell is the demonstration of clonality and the capacity to generate a myriad of functional cells. The ‘self-renewing’ attributes of a stem cell may manifest through symmetrical division, yielding exclusively either stem cells or all progenitor cells, or through asymmetrical division, capable of producing both stem cells and progenitor cells (Sanders et al., 2006).

### Sources of stem cells

**FIGURE 1:**
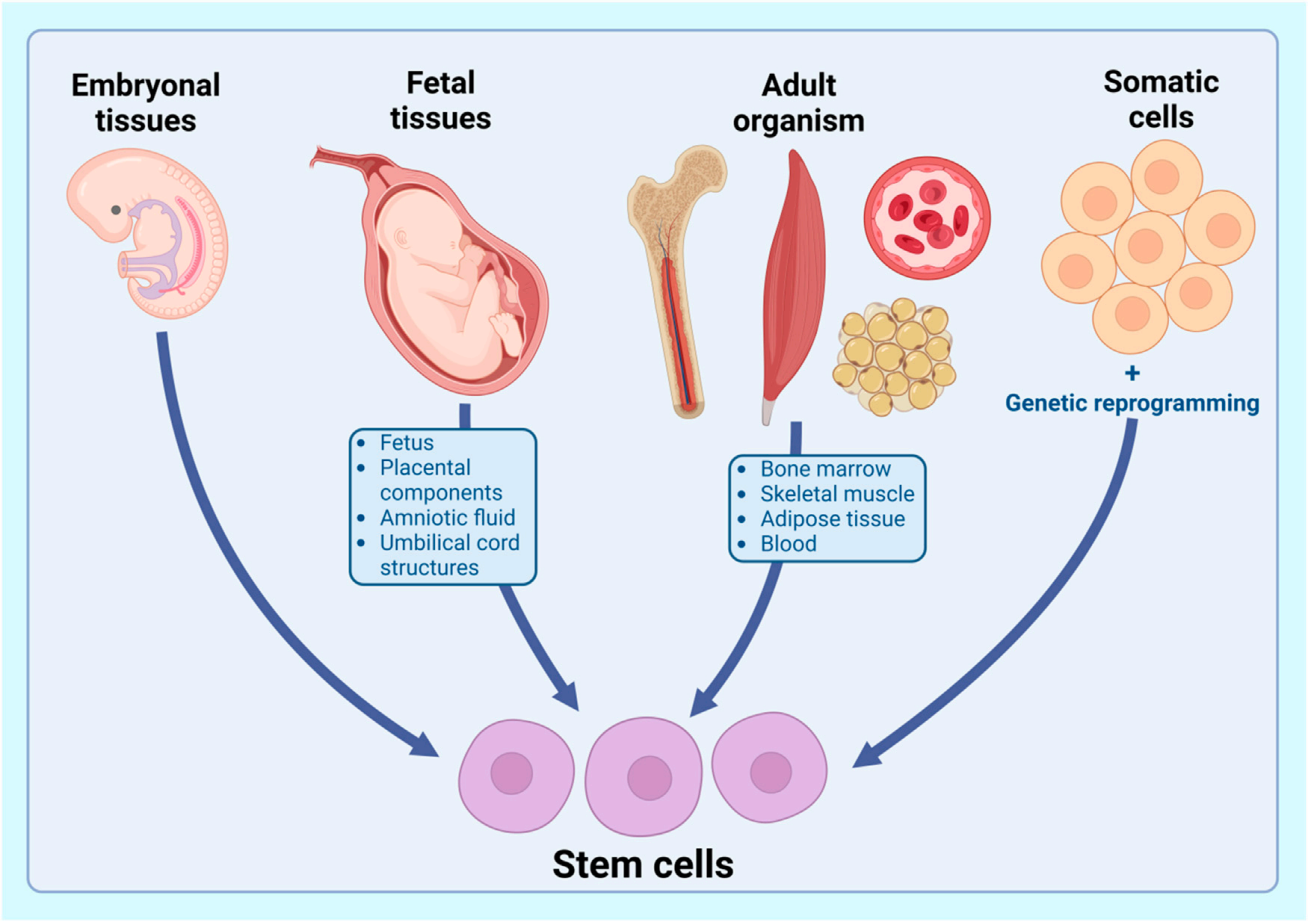
Sources of stem cells.

In the realm of regenerative medicine and cellular therapeutics, stem cells are procured from various biological reservoirs, encompassing four fundamental sources. These encompass (1) embryonic tissues, (2) fetal tissues such as the fetus, placental components (amnion and chorion), amniotic fluid, and umbilical cord structures (Wharton’s jelly and blood), (3) specific niches within the adult organism, notably adipose tissue, bone marrow, skeletal muscle, and blood, and (4) differentiated somatic cells subjected to genetic reprogramming, exemplified by induced pluripotent stem cells (iPSCs) (Figure 1.) (Bacakova et al., 2018; Mushahary et al., 2018; Lovell-Badge et al., 2021). Historically, bone marrow (BM) has stood as the preeminent reservoir of mesenchymal stem cells (MSCs) in the human context. Despite its richness in hematopoietic stem cells, BM harbors only a scant population of MSCs. Moreover, the arduous and anesthesia-requiring BM harvesting procedure has restricted the utilization of BM-derived MSCs (bmMSCs) in both investigative and clinical settings. Presently, MSCs are isolatable from diverse tissue sources, including dental tissues, integumentary structures, salivary glands, limb buds, and menstrual blood (Rodríguez-Fuentes et al., 2021). Adipose tissue (AT) emerges as a salient source of MSCs, characterized by its ubiquity and accessibility, rendering adipose-derived MSCs (adMSCs) pivotal candidates for autologous and allogeneic stem cell-based therapies and tissue engineering (Palumbo et al., 2018). In contradistinction, perinatal tissues, such as the amnion, chorion, and umbilical cord (UC), represent promising repositories of MSCs, given the advantageous attributes conferred by the youthfulness of the donors. While MSC derivation from various perinatal tissues is feasible, UC tissue, specifically Wharton’s jelly, emerges as a superior MSC reservoir when compared to umbilical cord blood (UCB) (Ferrúa et al., 2017; Chu et al., 2020).

The acquisition of stem cells is predicated upon two principal methodologies: enzymatic isolation and explant culturing. Enzymatic isolation procedures are contingent upon the source of MSCs, necessitating tailored techniques, specific media formulations, serum constituents, and cell surface markers (Bacakova et al., 2018; Mushahary et al., 2018). In the explant method, devoid of enzymatic intervention, the original tissue is sectioned into smaller fragments, subsequently arranged in culture dishes or flasks. After this, cells commence migration from the tissue fragments, adhering to the culture surface (Hendijani, 2017). The explant methodology is notable for its proclivity to generate cell populations characterized by reduced heterogeneity, resulting in augmented proliferation rates and enhanced cell viability, as compared to the enzymatic approach (Chu et al., 2020). This inclination is likely attributable to the presence of integral tissue fragments and undissociated extracellular matrix (ECM) during the explant culture process. These components contribute to the establishment of a protective milieu, mitigating the impact of proteolytic and mechanical stressors and thereby creating an environment conducive to the migration of cells (Sajeesh et al., 2020). While enzymatic digestion facilitates the isolation of fibroblast-like (stem) cells and the concomitant liberation of endothelial cells and pericytes, the explant culture paradigm offers additional advantages. These include the release of cytokines and growth factors into the culture medium, an augmented yield of stromal cells, a truncated proliferation timeframe, and the simultaneous expression of surface markers CD73, CD90, and CD105, coupled with the conspicuous absence of CD14, CD31, CD34, and CD45 in all MSC populations (Lu et al., 2021). Quantitative assessments predicated on nucleated cell numbers per unit tissue weight underscore that the explant culture methodology confers a superior yield of stromal cells in comparison to the enzymatic method. This observed advantage may stem from diminished cellular adhesion following exposure to enzymatic treatment and the concomitant loss of cells during the sequential procedures of filtration and washing inherent in the enzymatic methodology (Priya et al., 2014). The amalgamation of these methodologies underscores a nuanced approach to establishing reliable, robust, and standardized MSC isolation protocols, exemplifying the potential of UC and AT as highly relevant and promising tissue sources for MSCs (Mushahary et al., 2018).

Recent improvements in culturing hematopoietic stem cells (HSCs) focus on enhancing their *ex vivo* expansion and maintaining their functionality over extended periods. Advances include the development of fully defined, albumin-free culture systems that eliminate the variability of traditional supplements like bovine serum albumin. These new methods allow for substantial expansion of HSCs, with fold increases reaching several hundred times over a month. Improved culture conditions also support clonal analysis of HSC heterogeneity and enable transplantation into nonconditioned recipients, offering new potential for research and therapeutic applications in hematopoiesis and immune system studies (Wilkinson et al., 2020).

Current improvements in culturing mesenchymal stromal cells (MSCs) have focused on optimizing growth conditions to enhance their expansion, viability, and therapeutic potential. Key advancements include the use of serum-free or xeno-free media, which eliminates animal-derived components and reduces variability in culture outcomes. Newer bioreactor systems and 3D culture methods have also been developed, enabling more efficient large-scale expansion while preserving MSC functionality. Additionally, refined protocols for maintaining MSC stemness, differentiation potential, and immunomodulatory properties have contributed to their increased use in regenerative medicine and cell-based therapies (Todtenhaupt et al., 2023).

## The employment of stem cell culture in therapy and science (cell lines)

The growing interest in culturing stem cells excited many researchers all around the world to better understand the processes occurring in the cultures development and to predict the overall possibilities of these cells’ usage. Throughout recent years, many milestones have been reached in this field, and the use of stem cells cultures continues to grow and pins researchers’ faith on applying it in more treatment tactics and scientific research. Many research works are conducted using rodent stem cells (especially mouse ones), and the outcomes of these works suggest that cultured stem cells can help in the treatment of joints diseases (e.g., rheumatoid arthritis), peritonitis, colitis, and many more (Yen et al., 2023; Lan et al., 2021; Marega et al., 2023; Zaripova et al., 2023).

Therapeutic properties of stem cell cultures can be helpful for various specialists. Dierick et al. (2021) points out that multipotent stem cells (MSC) are currently under consideration as a possible treatment of pulmonary hypertension (Dierick et al., 2021). Jiang and Tuan (2023) in his work describes various types of stem cells used in creating a cartilage-like tissue in human joints, which improves the quality of life of patients affected with arthropathies (Jiang and Tuan, 2023; Giorgino et al., 2023; Hwang et al., 2021). One of the yet-to-overcome challenge remains employing stem cells to repair infarcted cardiac tissue. Cardiovascular diseases remain one of the most common deaths causes all around the world, and the possibility of heart regeneration is very promising (Parrotta et al., 2019; Campostrini et al., 2021; Barreto et al., 2019). The researchers are also examining the usage of cell lines, which are induced into male germ-like cells, in the treatment of male infertility (Malekmohamadi et al., 2019). Chen et al. (2020) in his work presents the idea of forming a healthy liver, using 3D stem cells, which would be transplanted to patients suffering from end-stage liver diseases, such as liver cancer (Chen et al., 2020). Scientists also utilize stem cells in the process of understanding mechanisms that occur in tumors, such as glioblastoma (Nikitin et al., 2023) and more.

It is worth underlining that the increasing knowledge regarding stem cell culture and its application in the modern therapeutic approaches is possible due to discovering new technologies, better understanding the processes taking place in stem cells, and the growing interest in molecular science. Apart from medicine, stem cells also find use in other sciences, such as ecology. Xie et al. (2020) in his work points out that obtaining these cells from various endangered fish species can help preserving them in the environment (Xie et al., 2020).

## The methods of culturing stem cells

The process of developing a stem cells culture has been improving and changing for several years. In 2006, the Japanese researchers, Takahashi and Yamanaka (2006), discovered how to turn a multipotent stem cell into a pluripotent one (Takahashi and Yamanaka, 2006). This has been one of the milestones in the stem cells culturing, as it enabled using these cells in human therapy, as the cells obtained in this process are biocompatible with the receiver.

**FIGURE 2:**
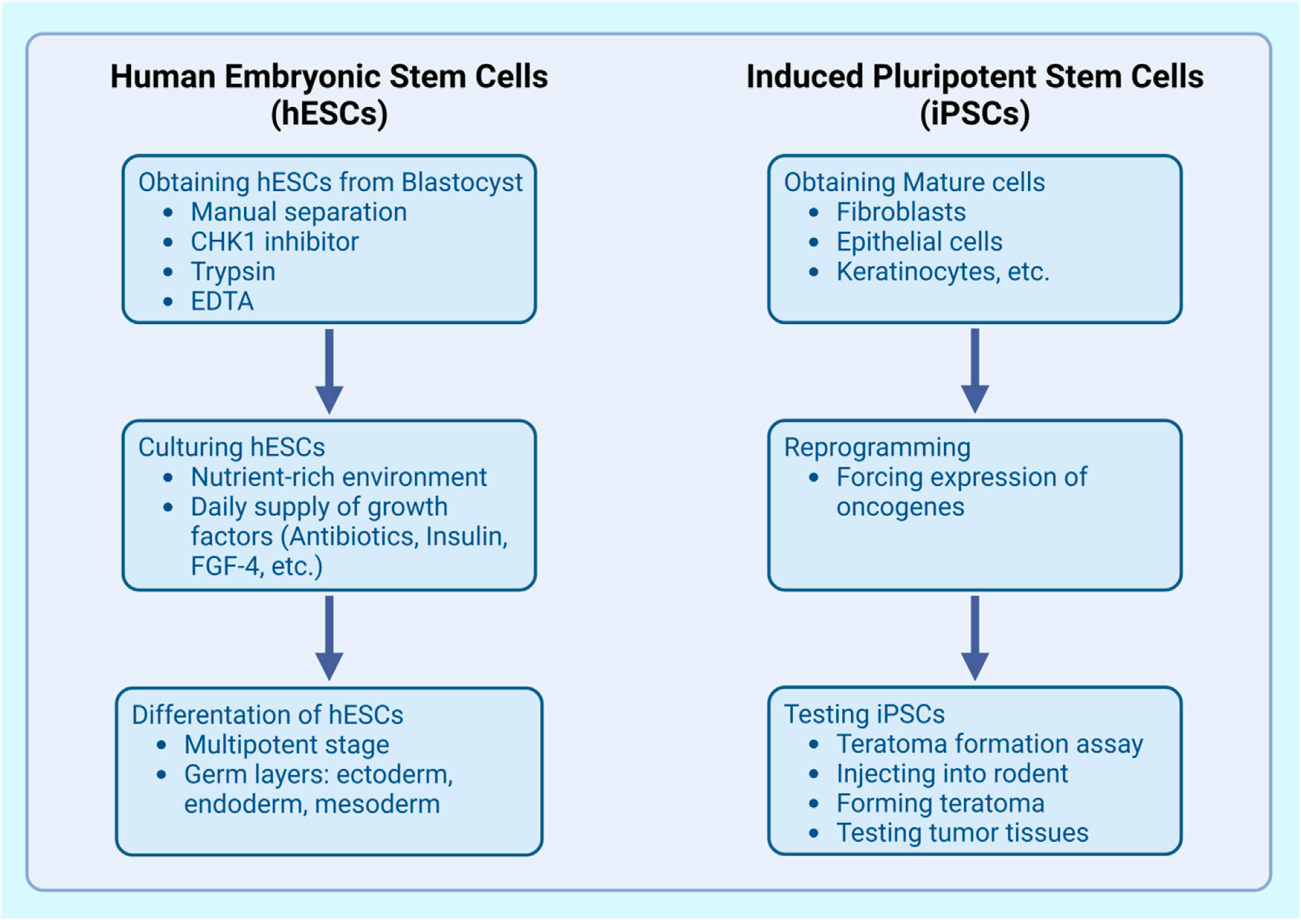
Stem cells culturing process.

There are several ways to culture stem cells, depending on the source of the cells themselves. In this work, there will be highlighted two methods of culturing stem cells (Figure 2.). Human embryonic stem cells (hESCs), the first type of stem cells, can be obtained from the preimplantation embryos, more precisely-the blastocyst (Zakrzewski et al., 2019). First, the hESCs need to be separated from the blastocyst (which can be done in the early stages of embryo differentiation, as it is safer for the embryo, or *in vitro*). There are several methods to perform this procedure. The simplest one is the manual separation. The other possibilities consist of using enzymes’ inhibitors, such as checkpoint kinase-1 (CHK1) inhibitor (Ware et al., 2023), trypsin or ethylenediaminetetraacetic acid (EDTA) (Zakrzewski et al., 2019). After the separation is done, the obtained cells need to be placed in a special environment, containing nutrients and growth factors, delivered daily. This mixture consists of such substances as antibiotics, insulin, transferrin, heparin, source of sodium, fibroblast growth factor-4 (FGF-4), and many more (Zhao et al., 2023). The culture will continue to grow, and with adequate factors, can be directed into more specified cells (Zakrzewski et al., 2019).

Moreover, the microenvironment plays a critical role in stem cell behavior, particularly in regulating stem cell fate. Factors such as extracellular matrix (ECM) composition, stiffness, and mechanical forces are key in determining the differentiation pathways of stem cells. For instance, variations in ECM stiffness can direct stem cells toward specific lineages; softer matrices tend to promote neurogenic differentiation, while stiffer matrices support osteogenic differentiation. Mechanical forces like wall shear stress and circumferential strain also significantly influence stem cell fate by modulating cellular signaling pathways and gene expression. These mechanical cues, in combination with the biochemical environment, create a complex microenvironment that ultimately governs stem cell behavior and differentiation (Discher et al., 2005; Engler et al., 2006).

After the differentiation, hESCs become multipotent, meaning that they can only develop into specified germ layer (ectoderm, endoderm, mesoderm). However, the earlier-mentioned discovery of Yamanaka and Takahashi allows to undo this process and acquire a pluripotent cell from a multipotent one. These stem cells are called induced pluripotent stem cells (iPSCs).

To obtain iPSCs, mature cells, such as fibroblasts, epithelial cells, keratinocytes et cetera are drawn from the organism (Zakrzewski et al., 2019). Later, these cells need to be reprogrammed by forcing an expression of oncogenes. To test whether obtained iPSCs have sufficient reproductive properties, a specific test needs to be conducted-the teratoma formation assay (Zakrzewski et al., 2019). In this test, the iPSCs are injected into a rodent, and start to form a teratoma. Further on, the tumor is tested to determine which types of tissues has it formed. The outcome shows how active they obtained iPSCs are. Some scientists also use the teratoma formation to work on the sourced tissues, after collecting the tumor from the rodent (Lee et al., 2020).

It is necessary to mention the efforts from Dr. Raimondi’s lab to develop faithful 3D models to preserve stem cell in a more physiological context (Donnaloja et al., 2020; Steimberg et al., 2020; Rey et al., 2020; Nava et al., 2012; Boeri et al., 2019). These models aim to mimic the natural stem cell niche more accurately than traditional 2D cultures, promoting more realistic cell behavior, differentiation, and self-renewal. Advances include the use of biomimetic scaffolds, hydrogels, and organoids that replicate the physical, chemical, and mechanical properties of the *in vivo* environment. These 3D systems improve the maintenance of stem cell stemness and functionality, providing more reliable platforms for studying cell biology, disease modeling, and potential therapeutic applications.

There is much yet to be discovered in culturing stem cells. Each year novel methods are discovered, and the therapeutic and research properties of stem cells are amplified.

## Problems and most common mistakes in cell culture management

Cell culture is a crucial technique in biological research, and proper management is essential to ensure reliable and reproducible results. Errors can occur in cell culture studies if they are not done correctly. To ensure the repeatability of *in vitro* research, it is crucial that cell culture investigations be carried out using good cell culture practice (GCCP) (Pamies et al., 2022). The abundance of erroneous and irreproducible results in the scientific literature is specifically caused by common problems in cell culture, such as cross-contamination between different species or within the same species, misidentification of cells, genetic drift, bacterial, fungal, yeast, viral, or chemical contamination, and the lack of strict quality control testing (Nelson-Rees and Flandermeyer, 1976). Rough estimates suggest that 16.1% of studies that were published used cell lines that had problems (Babic et al., 2019). It has been found that adding antibiotics and anti-mycotic to cell culture media helps reduce biological contamination from *mycoplasma*, yeast, bacterium, and fungus. These drugs usually work by preventing the formation of bacterial initiation complexes between mRNA and the bacterial ribosome (e.g., streptomycin), inhibiting the synthesis of cell walls (e.g., penicillin), or compromising membrane permeability (e.g., amphotericin B). However, prolonged usage of antibiotics may result in the sluggish growth rates of resistant or persistent bacterial pollutants, which may cause subtle alterations in cell behavior and differentiation (Llobet et al., 2015; American Type Culture Collection Standards Development Organization Workgroup ASN-0002, 2010). Therefore, to minimize bacterial contamination in cell culture, researchers should endeavor to maintain rigorous aseptic working conditions and refrain from using antibiotics in cell culture on a permanent basis (Ryu et al., 2017; Varghese et al., 2017). In cell cultures, contamination can come from chemical and biological sources. Indicators of this contamination typically include sluggish cell growth, morphological abnormalities, abrupt pH changes in the media, and higher concentrations of dead or floating cells in the culture. Regular screening for these pollutants in cell cultures is crucial to guarantee consistency in results and prevent potentially harmful consequences.

**Table:** 

Type	Characterization	Ref.
Viruses	Due to the lack of impact on cellular growth, detecting viral contamination can be challenging. The minute size of these viruses (approximately 100 nm in diameter) renders them invisible under a bright-field microscope. Although cytopathic virus infections may not influence cellular growth, they can still compromise the integrity of the culture. Furthermore, laboratory personnel working with virally infected cell cultures may be at risk of health hazards	Schroder et al. (2022), Gombold et al. (2014), Meten (2002), Geraghty et al. (2014), Borojevic et al. (1985)
Mycoplasmas	Mycoplasmas are spherical to filamentous cells that lack intracytoplasmic membranes and cell walls. With a diameter of about 300 nm, they are the smallest self-replicating organisms. *Mycoplasma* infections can alter various cellular activities of the host culture, including growth, metabolism, migration, morphology, and responsiveness to growth factors. Additionally, certain *mycoplasma* species may cause chromosomal damage and abnormalities in the data	Drexler and Uphoff (2002), Rottem, 2003; Into et al. (2004)
Bacteria	The sizes and shapes of bacteria vary greatly, ranging from 0.5 to 20 µm. In cell culture conditions, bacterial pollutants can spread and colonize rapidly. When tiny, moving granules appear between cells a few days after initial contamination, microscopy is typically able to detect such contamination	Taur et al. (2014), Bartfeld et al. (2015)
Yeast and mold	Fungi, including yeast cells, multiply more quickly than cells found in mammals. They are typically 3–4 µm in size, although they can grow up to 40 µm. Within two to 3 days, contamination can be easily detected by microscopic analysis or by changing the medium’s color. Notably, yeast is not harmed by antibiotics such as streptomycin and penicillin	Puerta-Alcalde and Garcia-Vidal (2021), Otto and Green (2020)
Parasites	Certain precautions may need to be taken when handling freshly generated primary cell cultures from a donor organism that is suspected or confirmed to be infected with intracellular protozoan parasites (such as *Toxoplasma gondii, Trypanosoma cruzi*, *Leishmania* spp.*, Cryptosporidium parvum, or Plasmodium* spp.). It is important to take additional safety measures, such as wearing protective gear and clothes. Needles and other sharp things should not be used when working with parasite-infected cell lines to reduce potential dangers	Herwaldt (2001), Herbert et al. (1980)
Prions	Prions are difficult to inactivate since they are primarily made up of the protein PrPSc and lack nucleic acids. Although most cell lines are resistant to prion infection, some susceptible cell lines allow prions to replicate steadily and permanently. These prions could be added to cell culture medium that has been supplemented with bovine serum. Interestingly, prions are notoriously hard to inactivate	Avar et al. (2020), Chou et al. (2015)
Chemical, biological, and other nonliving contaminants	Optimal cell growth can be negatively impacted by endotoxins/lipopolysaccharides, detergents, metals, hormones, growth factors, disinfection and cleaning agent residues, plasticizers, and other contaminants. Water, sera, contaminated reagents, and specific culture supplements can all lead to chemical contamination. Cell cultures might also be jeopardized by impurities such as detergent residues or residues on storage containers, glasses, pipettes, or tools used during disinfection. Plasticizers may leak out of storage bottles and plastic tubing. Prolonged exposure to visible or fluorescent light can produce free radicals by photoactivating substances like riboflavin, tryptophan, or buffering agents like HEPES and PIPES.	Nims and Price (2017), Grzelak et al. (2001), Zigler et al. (1985)
Inter- and intra-species cross-contamination	Cross-contamination in cell lines is both frequent and substantial. Inter- and intra-species cross-contamination can originate from various sources, including aerosol transmission, the use of unfiltered pipettes, shared media and reagents between different cell lines, and the application of conditioned materials	Capes-Davis et al. (2010), Huang et al. (2017), Weiskirchen (2022)

Different cell types may require specific culture vessel coatings or substrates for optimal attachment and growth. Using inappropriate surfaces can result in poor cell adhesion and viability. Primary cells can be more sensitive than established cell lines. Attention must also be paid to specific requirements, such as shorter passage intervals, specialized media, and unique culture conditions.

In biomedical research, cell culture is essential, but dependability is compromised by problems including contamination and misidentification. Authentication is aided by resources such as International Cell Line Authentication Committee (ICLAC) and required testing. Adopting best practices, training employees, and utilizing modern innovations like patient-derived organoids (PDOs) and patient-derived xenograft (PDXs) are all necessary to ensure safety. Reliance on animals is decreased by the rapid production of patient-derived cell cultures made possible by CRISPR technology. Continuous training of personnel is essential for a dependable cell culture facility.

## Decellularized extracellular matrix

Decellularized extracellular matrix (dECM) derived from cells has emerged as a powerful tool in stem cell culture, offering a variety of benefits that enhance the growth, differentiation, and overall functionality of stem cells. The dECM is a scaffold composed of proteins, glycoproteins, and other components that mimic the natural extracellular environment, providing crucial signals and structural support to cultured cells. Here are the key benefits of using cell-derived dECM in stem cell culture (Wang et al., 2021b; Pei, 2017; Kim et al., 2018; Yao et al., 2019; Zhang et al., 2021):

1. Mimicking the Native Microenvironment
  • Biochemical Cues: The dECM retains bioactive molecules from the original tissue, which can provide essential biochemical cues that guide stem cell behavior, including proliferation, differentiation, and migration. These cues are difficult to replicate using synthetic materials.
  • Structural Support: The natural architecture of dECM offers a physical framework that closely resembles the *in vivo* environment, supporting proper cell attachment, organization, and growth.
2. Enhanced Stem Cell Differentiation
  • Tissue-Specific Differentiation: dECM derived from specific tissues can induce lineage-specific differentiation of stem cells. For instance, cardiac dECM can promote cardiac lineage differentiation, while bone dECM can enhance osteogenic differentiation.
  • Regulation of Stemness: The presence of specific ECM components can maintain the balance between stem cell self-renewal and differentiation, depending on the culture conditions and the type of dECM used.
3. Improved Cell Viability and Proliferation
  • Reduced Apoptosis: The bioactive environment provided by dECM reduces cell stress and apoptosis, leading to higher cell viability during culture.
  • Proliferative Support: The natural composition of dECM can stimulate cell proliferation more effectively than traditional culture substrates, allowing for the expansion of stem cell populations.
4. Immunomodulatory Effects
  • Immune Compatibility: dECM derived from autologous or allogeneic cells can reduce the immune response in regenerative medicine applications, which is critical for the survival and integration of transplanted stem cells.
  • Anti-inflammatory Properties: Some dECM components possess inherent anti-inflammatory properties, which can help in maintaining a conducive environment for stem cell growth and function.
5. Customization and Versatility
  • Source Flexibility: dECM can be derived from various tissues, enabling customization for specific stem cell applications, such as neural, cardiac, or musculoskeletal tissues.
  • Combinatorial Approaches: dECM can be combined with growth factors, cytokines, or other bioactive molecules to further tailor the microenvironment for specific stem cell needs.
6. Facilitating 3D Culture Systems
  • 3D Scaffold Formation: dECM can be used to create three-dimensional (3D) culture systems that more accurately mimic the *in vivo* conditions, which is crucial for studying cell behavior in a more realistic context.
  • Tissue Engineering: The use of dECM in 3D culture systems facilitates the development of complex tissue constructs, supporting the growth of stem cells in a manner that promotes tissue regeneration and repair.
7. Reduction in Use of Animal-Derived Products
  • Xeno-Free Cultures: dECM provides a viable alternative to animal-derived matrices, such as Matrigel, in creating xeno-free culture environments, which is especially important for clinical and translational applications.
8. Potential for High-Throughput Screening
  • Scalability: dECM can be processed and applied in high-throughput screening platforms, allowing for the testing of various conditions and compounds on stem cells in a controlled and reproducible manner.

In summary, cell-derived dECM offers a multitude of benefits for stem cell culture, including the provision of a native-like microenvironment, enhanced differentiation and proliferation, and the ability to customize the scaffold for specific applications. These advantages make dECM a valuable tool in advancing stem cell research, regenerative medicine, and tissue engineering.

## Discussion

The article’s examination of stem cell cultivation techniques is more than just a summary of recent developments; it's a story that spans decades of cellular biology’s evolutionary landscape and captures decades of scientific research, invention, and hope. Stem cells represent the unfathomable complexities of nature and provide insight into the regeneration potential hidden in all living things. They are frequently hailed as the cornerstone of regenerative medicine (Daley, 2012; Engle and Puppala, 2013).

The essay takes readers on a tour through the history of stem cell research, starting with the seminal findings of Takahashi and Yamanaka, whose ground-breaking work revealed the revolutionary idea of induced pluripotent stem cells (iPSCs). Because iPSCs can be derived from somatic cells and yet have the pluripotency of embryonic stem cells, they have not only changed the ethical landscape of stem cell research but have also made cellular reprogramming more accessible to the public. This has opened new avenues for personalized medicine and disease modeling (Nonaka et al., 2004).

During the acknowledgment of these paradigm-shifting discoveries, the piece also elucidates the complex interplay between scientific advancement and inherent challenges. Stem cell cultivation, with its intricate dynamics, presents significant obstacles for researchers. A primary concern is the genetic instability observed in long-term cultures, which necessitates a meticulous balance between preservation and expansion. Monitoring genetic stability is crucial to ensure that stem cells maintain their intended characteristics and avoid accumulating deleterious mutations. Due to the inherent heterogeneity within stem cell populations—comparable to a painter’s palette—novel methodologies are required to achieve uniformity. Such homogeneity is essential for effective therapeutic applications, as consistent genetic profiles are vital for reliable and successful treatments (Ben-David, 2015; Briu et al., 2021; Turinetto et al., 2017).

The essay lays out a path towards the horizon of possibility in response to these difficulties, where cutting-edge innovations like machine learning and artificial intelligence (AI) serve as sentinels, pointing the way forward. AI integration offers a symbiotic evolution where human creativity and data-driven insights combine to create previously unimaginable levels of efficiency and reproducibility. This is in addition to optimization (Altyar et al., 2023; Nosrati et al., 2021).

Furthermore, the conversation touches on a wide range of scientific topics, going beyond the boundaries of medicinal applications. Like the crucibles of alchemists, stem cell cultures are at the crossroads of several fields, ranging from ecological conservation to pulmonary biology. In addition to providing a window into the secrets of disease pathology, they hold the key to restoring the delicate balance of ecosystems, conserving endangered species, and opening the door to nature’s resilience (Ponti et al., 2005; Todaro et al., 2007; Eramo et al., 2008; Fischer et al., 2015).

However, amidst the grandeur of scientific endeavor, the article grounds itself in the sobering reality of laboratory practice. It underscores the paramount importance of meticulous cell culture management, where the pursuit of reliability and reproducibility stands as a sentinel against the specter of contamination and misidentification. Strategies ranging from stringent aseptic technique. The essay, however, firmly roots itself in the grim realities of laboratory work, even amid the magnificence of scientific achievement. It emphasizes how crucial it is to handle cell cultures meticulously, with the goal of reproducibility and dependability acting as a guard against contamination and misidentification. Techniques varying from strict aseptic methods to prudent use of antibiotics serve as the barrier against the incursion of biological whims, guaranteeing that the foundation of scientific investigation is integrity (Smith et al., 2014; Luizon et al., 2016).

Finally, the paper becomes more than just a discussion; it becomes a symphony that speaks to the goals, victories, and struggles that the scientific community shares. It is evidence of the tenacious spirit of inquiry, the unrelenting search for truth, and the unshakable faith in the revolutionary potential of stem cell research to show the way towards a more promising and health-conscious future for everybody.

## Conclusion

The paper explores advancements in stem cell research, covering methods, challenges, and perspectives. It discusses breakthroughs like induced pluripotent stem cells (iPSCs) and hurdles such as genetic instability. It also highlights the potential of stem cell-derived products in personalized therapies. The section on stem cell origins includes perinatal tissues and induced pluripotent stem cells (iPSCs), detailing acquisition methods and their benefits. It emphasizes the importance of technology and molecular research in improving stem cell culture. The article discusses techniques for generating iPSCs and human embryonic stem cells (hESCs) and addresses common issues in cell culture management.

- iPSCs and hESCs present promising avenues for research and therapy;
- Genetic instability poses a significant challenge in stem cell applications;
- Stem cell-derived products hold immense potential for personalized medicine;
- Perinatal tissues and iPSCs offer accessible sources for stem cell research;
- Technological advancements and molecular insights are crucial for enhancing stem cell culture;
- Training, best practices, and cutting-edge technology are imperative for progress in biomedical research.

Overall, the paper underscores the critical role of stem cell culture in advancing medicine and emphasizes the importance of addressing key challenges and leveraging emerging opportunities in the field.
